# Thermal Degradation Kinetics Analysis of Ethylene-Propylene Copolymer and EP-1-Hexene Terpolymer

**DOI:** 10.3390/polym14030634

**Published:** 2022-02-07

**Authors:** Hassam Mazhar, Farrukh Shehzad, Sung-Gil Hong, Mamdouh A. Al-Harthi

**Affiliations:** 1 Department of Chemical Engineering, King Fahd University of Petroleum & Minerals, Dhahran 31261, Saudi Arabia; hassam.mazharulhaque@kfupm.edu.sa (H.M.); farrukh@kfupm.edu.sa (F.S.); 2 TS&D Center, S-OIL Corporation, Seoul 07793, Korea; sghong0927soil@gmail.com; 3 Center for Refining and Advance Chemicals, The Research Institute, King Fahd University of Petroleum & Minerals, Dhahran 31261, Saudi Arabia

**Keywords:** ethylene-propylene copolymer, ethylene-propylene-1-Hexene terpolymer, polymer nanocomposite, polymerization, layered double hydroxides (LDH), thermal analysis, degradation kinetics, master plots

## Abstract

LLDPE is a less crystalline polymer with vast industrial and domestic applications. It is imperative to understand the synthesis, processing conditions, and thermal degradation mechanism of the co- as well as terpolymers. This paper reports the in-situ synthesis and thermal degradation studies of the ethylene-propylene copolymer and ethylene-propylene-1-hexene terpolymer and its nanocomposite with ZnAL LDH sheets. The 1-hexene dosing during the in-situ process influenced the product yield and immensely affected the thermal stability of the resultant polymer. One milliliter 1-hexene in-situ addition increased the product yield by 170 percent, while the temperature at 10 percent weight loss in TGA was dropped by about 60 °C. While only 0.3 weight percent ZnAL LDH addition in the terpolymer improved the thermal stability by 10 °C. A master plot technique and combined kinetics analysis (CKA) were deployed to access the thermal degradation mechanism of the synthesized polymers.

## 1. Introduction

The polymerization of ethylene and ethylene with α-olefins using a class of metallocene catalysts delivers very high catalytic activity and precise control over the stereo-regular properties of the resultant polymer [1]. The ethylene-propylene (EP) copolymer and ethylene-propylene-α-olefins terpolymers are versatile polymeric materials with properties ranging from thermoplastic to elastomers [2]. Unlike the conventional Ziegler-Natta catalysts the metallocene catalysts are homogenous and produce high polymer yield with a low polydispersity index (PDI). Despite the advances in properties of polymers synthesized through metallocene homogenous catalysts, these polymers present certain limitations for applications requiring higher thermal and mechanical stability. It is desired to synthesize polyolefin with improved thermal stability [3], mechanical properties, decreased flammability [4], and gas permeability [5]. The properties of these polymers can be further improved by the incorporation of nanomaterials in the polymer matrices to form a polymer nanocomposite. Such polymer nanocomposites exhibit improved mechanical and thermal properties [6].

Over the past two decades, the synthesis of polymer nanocomposites has garnered immense research interest. The focus is to improve the properties of different polymers such as improvement in thermomechanical stability or improvement towards specific applications such as water purification, packaging, and biomedical applications [7,8,9,10]. Similarly, enhancements in electrical and thermal conductivity by various nanofillers, such as carbon nanotubes and graphene have also been demonstrated. The polymer nanocomposites offer superior physical and mechanical properties in comparison to the neat polymer, partially because of the prevailing large interfacial area between the nanofillers and the host polymer. Graphene [11,12], hexagonal boron nitride (hBN) [2], titania [13], and layered double hydroxides (LDHs) [12,14], etc., are a few classes of 2D nanomaterial being extensively used in the synthesis of the polymer nanocomposites. These nanomaterials inherent novel properties such as mechanical, chemical, thermal, optical properties, etc., attributes these materials could contribute to the staggering properties of the resulting polymer nanocomposites. The composites of the nanosheets with the polymers had exhibited advancement in the thermal and mechanical properties. The extended use of polymers is applicable by the formation of low-cost polymer nanocomposites which can serve as an alternative to materials such as metals, glass, ceramics, etc. [15,16,17]. Polyolefin nanocomposites are assumed to be the next innovation with potential physical and mechanical properties [18].

However, it is challenging to completely translate the properties of nanomaterial to the resulting polymer composites. The properties of the composites majorly depend on the functionalization of the nanomaterials and the methodology of its incorporation in the polymer matrix. The features of a composite are determined by the degree of dispersion of the nanosheet in the polymer matrix, therefore it is crucial to achieve uniform dispersion of the nanosheet within the polymer matrix for a synergistic effect of the nanomaterial. They are incorporated in the polymer matrix through (1) Melt blending, (2) Solution casting, and (3) in-situ polymerization. Among the mentioned methodologies in-situ polymerization delivers better dispersion caused by exfoliation [19].

Graphene, Carbon nanotubes and sheets, LDHs, and other classes of 2D nanosheets have been reported as nanofillers in polymer (PE, PMMA, PS, etc.) nanocomposites. Carbon-based nanocomposites of polyolefins have been studied extensively as reported by many researchers [3,20,21,22]. The main attributes of these nanomaterials incorporated through in-situ polymerization influence the catalytic activity, polymer morphology, and molecular weight distribution (MWD). In particular, LDHs are of great interest because of their chemical and mechanical stability, low cost, easy synthesis, and tailored properties. The versatile nature of the double layers applies to any specific requirements [12].

LDHs are a class of lamellar compounds comprising of the cationic brucite-like layers with intercalated charge compensating anions. The general formula of an LDH may be formulated as $\left[M_{1-x}^{2+}M_{x}^{3+}{\left(\mathrm{OH}\right)}_{2}\right]\;{\left[A^{n-}\right]}_{x/n}.{\mathrm{zH}}_{2}O\;$ where the value of *x* lies between 0.2–0.33, $M^{+2}$ and $M^{+3}$ can be divalent and trivalent metal cations, respectively, and $A^{n-}$ can be a variety of anions, such as ${\mathrm{CO}}_{3}^{2-}$, ${\mathrm{NO}}_{3}^{-}$ and ${\mathrm{SO}}_{4}^{2-}$. Over the past two decades, these layered nanosheets have attracted researchers’ attention to develop an inorganic-organic host-guest nanocomposite of desired chemical and physical characteristics, which renders enchanted control over the rate of reaction, product distribution, and stereochemistry. LDHs are highly tenable in terms of composition which caters to abundant choice of intercalation anion opens up a new perspective of functional material with novel properties [23].

LDHs have been used as nanofillers [24,25] and as catalyst support [20,26,27,28,29] in the polymer nanocomposites (polyethylene-PE, Polypropylene-PP) by in-situ polymerization. LDHs influence the MWD, crystallinity, polymer morphology, catalytic activities and improve flame retardancy.

LDH has been used as a support for Zeigler-Natta and metallocene catalyst for ethylene polymerization and it has also been applied as a filler in various polymers [24,30,31,32]. These studies reported that the presence of different types of LDH in the polymer (polyethylene PE, polypropylene PP) matrix affects the catalytic activity, molecular weight distribution, polymer, morphology, and crystallinity. Consequently, LDHs influence the physical and chemical properties of the polymer. PE/PP or polyethylene-polypropylene (EP) are semicrystalline polymers with enough constituent long chains and undergo crystallization under suitable conditions. Gao et al. [24] in their studies reported that the thermal stability of HDPE/ZnAL LDH composites was increased by 67 °C at 50% weight loss. The flame retardant property was also enhanced by the incorporation of the LDH. Naffer et al. used 5% by weight of a ternary LDH as filler to protect the LDPE from UV degradation [22].

Despite the advances of the polyolefins/LDH nanocomposites, it is crucial to investigate the thermal stability and degradation kinetics of this material for high-temperature applications. The composites are scrutinized under different thermal conditions to understand the thermal degradation kinetics. Accurate kinetic analysis requires the estimation of three terms (a) Activation energy (Ea), (b) Pre-exponential factor A, (c) Kinetic model *f*(*α*). Where *f*(*α*) is also known as the conversion function which imitates the degradation kinetics of a sample into a physical kinetic model. The degradation kinetics of polymers has been studied by many researchers, most of them have a consensus that polymer nanocomposites followed the first or nth-order model [33] and model-free method [34,35] of kinetic degradation. Our research team has also reported thermal degradation studies for polyethylene [36,37,38] and microwave irradiated polymers [3]. Nonetheless, few researchers partly agree with the concept and reported that the kinetics is not just controlled by the first or nth-order model and model-free method but is predominantly regulated by the random scission or diffusion mechanism [34,35,36,37,38,39]. Therefore, it is important to study the thermal degradation kinetics of *co*- and *ter*polymer and their nanocomposites rather than relying on previous studies.

This paper reports the synthesis and thermal degradation kinetics studies of the α-olefin copolymer, terpolymer, and terpolymer/LDH nanocomposite. The in-situ polymerization of ethylene-propylene and 1-Hexene (EPH) was performed using a simple metallocene catalyst. Additionally, the EPH/LDH nanocomposites were synthesized through the same route. Moreover, this paper discusses the influence of the in-situ addition of 1-hexene and LDH over the product yield and thermal behavior of the EPH/LDH. The thermal stability data obtained from thermogravimetric analysis (TGA) were utilized to evaluate the activation energy (Ea) by deploying the differential isoconversional method. The master plot technique and combined-kinetic analysis (CKA) were employed to predict the most suitable degradation kinetic model.

## 2. Experimental

### 2.1. Chemical Reagents

Ethylene-propylene (95:05) gas mixture (99% pure) purchased from Igas (Dammam, Saudi Arabia). 1-hexene, Bis(cyclopentadienyl) Zirconium(IV)dichloride, Bis(ter-methylcyclopentadienyl) Zirconium(IV) dichloride, modified methylaluminoxane (MAO), Zinc nitrate hexahydrate [Ni(NO_3_)_2_•6H_2_O], Aluminium nitrate nonahydrate [Fe(NO_3_)_3_•9H_2_O], sodium dodecyl sulfate, toluene, ethanol, methanol and all other chemicals were purchased from Sigma Aldrich (Darmstadt, Germany).

### 2.2. Polymerization

The in-situ polymerization reaction of EPH and EPH/LDH was carried out in a 250 mL Schlenk flask reactor (Sigma Aldrich, Darmstadt, Germany) under the vigorous stirring condition at temperature 60 °C and feed gas pressure 5 psi. Initially, the flask is fed with 6 mg of Zr catalyst, 15 mg LDH, and 80 mL toluene solvent inside the glove box. The sealed flask is taken out from the glove box. Nitrogen gas initially present in the flask is evacuated through a vacuum pump followed by feed gas (ethylene-propylene gas with 95:05 molar ratio) saturation. After 1-min certain amount of 1-hexene was injected (applicable only for terpolymer synthesis). After 3 min of feed gas saturation in the toluene solvent, 5 mL MMAO co-catalyst was injected into the reactor to initiate the polymerization reaction. The reaction was carried out for 30 min. Then, 100 mL methanol containing HCl (5% by volume) was introduced to the flask under vigorous stirring to quench the reaction for 45 min. The product was filtered off under simultaneous washing with excess methanol, the filtrate was kept for drying in an oven at 40 °C for 16 h to obtain the final polymer product [37].

### 2.3. FTIR

The FTIR analysis of the LDH sample was analyzed with a Nicolet-6700 Fourier Transform infrared (FTIR) (Thermo Electron Scientific Instruments Corp, Madison, WI USA). The FTIR scans were performed in a range of wavenumber 500 to 4000 cm^−1^ in attenuated total reflection (ATR) mode.

### 2.4. Thermal Characterization of Polymer

Differential scanning calorimetry (DSC Q-1000, TA Instruments, New Castle, DE, USA) was used for the thermal characterization of the polymer samples in an inert environment. About 5 mg of the polymer samples were loaded in an aluminum pan, which was subjected to a heat-cool-heat cycle in a temperature range of 30–160 °C at a heating and cooling rate of 10 °C/min each. The peak that appeared in the cooling and heating cycles gives the value of crystallization (T_c_) and melting (T_m_) temperature of the polymer, respectively. The integral area of the curves in each cycle can be used to estimate the crystallinity of the sample.

The thermal stability of samples was studied by the TGA (SDT-Q1000, TA Instruments, New Castle, DE, USA). TGA instrument was operated in a temperature range of 30–800 °C at various heating rates (5, 10, 15, 20 °C/min) under an inert environment. The values of activation energy were estimated by the isoconversional method upon employing the thermal degradation data obtained from the TGA operated at different heating rates. These studies contribute to envisaging the most appropriated degradation mechanism by the master plot technique. TGA provides weight loss of the sample with time, which is converted to fractional conversion (*α*) [40], by the following formula:

(1) $$\alpha =\frac{m_{i}-m_{t}}{m_{i}-m_{\infty }}$$

where *m_i_*, *m*_∞,_ and *m_t_* are initial, final, and instantaneous weight of sample, respectively. The factional conversion evaluated with respect to temperature, employed to estimate the activation energy by isoconversional method. For thermal degradation of a polymer, a general form of solid-state reaction rate is defined in terms of fractional conversion with respect to time, reaction model *f*(*α*), and rate constant.

(2) $$\frac{d\alpha }{dt}=kf\left(\alpha \right)$$

For temperature ramp rate *β*, temperature and time are in a linear relationship. Where *T_o_* and *T* are the initial and instantaneous temperature, respectively.

(3) $$T=T_{o}+\beta t$$

The rate expression for non-isothermal conversion in differential form can be expressed by a combination of Equations (1) and (2) as below equation [41]:

(4) $$\frac{d\alpha }{\mathrm{dT}}=\frac{k}{\beta }f\left(\alpha \right)$$

where *k* is a non-isothermal reaction rate constant which can be expressed in Arrhenius form as below.

(5) $$k=Ae^{\left(\frac{-E_{A}}{RT}\right)}$$

The activation energy of the degradation reaction is evaluated by model-free or model-fitting methods. Thermal degradation of the polymer involves the change of physical state with time or temperature; therefore the activation energy value is not constant throughout the process. However, the model-fitting method assumes a constant activation energy value which might lead to erroneous results [42]. While the isoconversional or model-free method does not require the assumption of the kinetic model but uses multiple heating rates data [11,42,43]. This method may facilitate a comprehensive insight into the reaction progress and temperature dependence. The model-free method is preferred over the model-fitting method considering its accuracy. The activation energy for a solid-state reaction can be evaluated by the differential Friedman method [44] by employing the rate of change fractional conversion with temperature data obtained at various heating rates (*β*). The slope of the linear regression of the data in the Friedman equation (Equation (6)) at a given constant value of α, gives the value of activation energy.

(6) $$\ln{\left(\beta_{i}\left(\frac{d\alpha }{dT}\right)\right)}_{\alpha }=-\left[lnf\left(\alpha \right)+\frac{E_{A}}{RT_{i}}\right]$$

The thermal degradation reaction mechanism of a polymer can be well understood by a suitable reaction model, which helps to maintain the processing conditions for a polymer. Table 1 shows a list of standard reaction models based on the reaction mechanism in a differential form. The regression of the experimental reaction data with the standard models determines the followed reaction mechanism and so the reaction model. A theoretical master plot following standard reaction models is deployed to determine an appropriate reaction model during the degradation reaction. The methodology does not require kinetic data such as activation energy (Ea) and frequency factor. The goodness of the fit of the experimental data in the listed models is assumed to follow the corresponding mechanism.

Generalized master plots are based on non-dimensional entity fractional conversion (*α*) and generalized time (*θ*). The differential form of the generalized time (*θ*) with respect to the time of reaction is given as [45,46].

(7) $$\frac{d\theta }{dt}=e^{\left(\frac{-E_{A}}{RT}\right)}$$

By combining Equations (4), (5), and (7) we get:

(8) $$\frac{d\alpha }{d\theta }=kf\left(\alpha \right)$$

(9) $$\frac{d\alpha }{d\theta }=\frac{d\alpha }{dt}e^{\left(\frac{-E_{A}}{RT}\right)}$$

Equation (9) is normalized at *α* = 0.5 to get the following form of generalized master plot equation.

(10) $$\frac{\frac{d\alpha }{d\theta }}{{\frac{d\alpha }{d\theta }}_{0.5}}=\frac{f\left(\alpha \right)}{f\left(0.5\right)}=\left(\frac{\frac{d\alpha }{dt}}{{\frac{d\alpha }{dt}}_{0.5}}\right)\frac{Exp\left[\frac{-E_{A}}{RT}\right]}{Exp\left[\frac{-E_{A}}{RT_{0.5}}\right]}$$

$\frac{f\left(\alpha \right)}{f\left(0.5\right)}\;$ in the above equation indicates the theoretical term calculated based on the models listed in Table 1. Whereas the experimental data are substituted in the term right side of the equal sign. The experimental and the theoretical models are overlapped in the master plot. The best fit is an idea about the kinetic model being followed during the degradation reaction.

However, a combined kinetic analysis (CKA) accesses the goodness of regression analysis of a particular kinetic model. The combination of Equations (2), (4), and (5) yields the CKA equation (Equation (11)). The slope and intercept of the CKA straight line give the values of the activation energy and preexponential factor, respectively.

(11) $$Ln\left(\frac{\beta \frac{d\alpha }{dT}}{f\left(\alpha \right)}\right)=Ln\left(A\right)-\frac{E_{a}}{RT}$$

## 3. Result and Discussion

### 3.1. LDH Characterization

**Figure 1:**
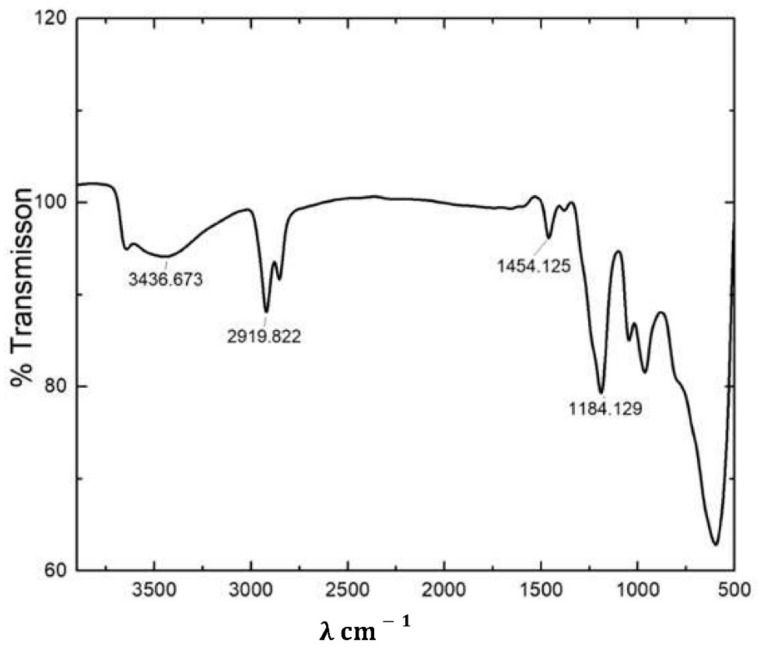
FTIR spectra of ZnAl-DDS LDH.

The ZnAl-DDS LDH was characterized by FTIR. A strong and broad peak centered at 3436 cm^−1^ was observed for ZnAl-DDS LDH (Figure 1). The peak at 3436 cm^−1^ corresponds to the stretching vibration of the hydroxyl group present in the LDH. Intense peaks observed at 2919 and 2858 cm^−1^ correspond to the C-H band stretching of the CH_3_/CH_2_ group belonging to the dodecyl sulfate molecules intercalated in the galleries of the LDH. A weak peak that appeared near 1600 cm^−1^ relates to the bending vibration of interlayer water molecules. The peak at 1454 can be assigned to C-H bending vibration [47]. The peak at 1184 cm^−1^ can be assigned for S=O bond stretching. Similar FTIR peaks for ZnAl-DDS LDH have been reported by Yaun et al. [2].

### 3.2. Yield

**Figure 2:**
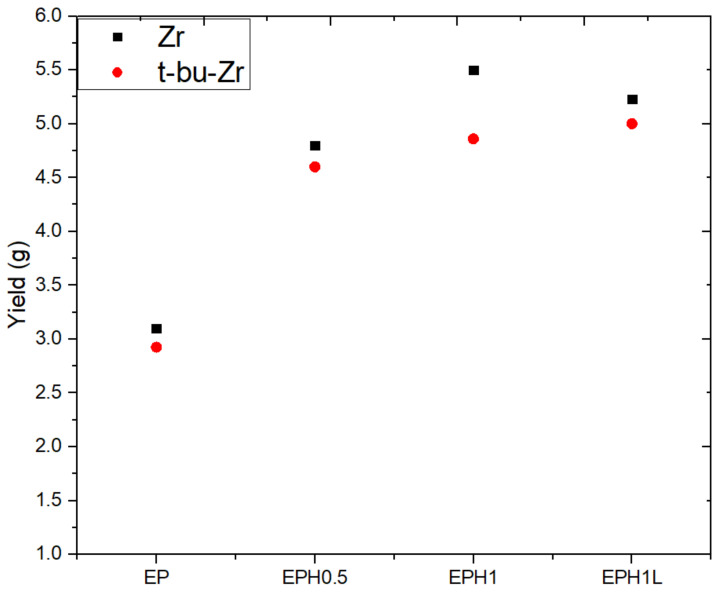
The plot shows the average yield of respective repeated samples. The ‘black square’ represents polymer yield synthesized with Zr catalyst, while the ‘red dot’ represents the yield corresponding to t-bu-Zr catalyzed polymer products. The reported yield is an average of a minimum of two concurrent polymer product yields.

The polymerization reaction was performed using two different catalysts as mentioned in Table 2. EP, a terpolymer of EP with 0.5 mL 1-hexene, EP with 1 mL 1-Hexene, and EP with 1 mL 1-Hexene and 15 mg NiFe LDHs were named as EP, EPH0.5, EPH1, and EPH1L, respectively (see Table 2). A very interesting trend in the product yield was observed. The EP polymer yield was increased with the addition of a small amount of 1-hexene. Initially, 0.5 mL 1-hexene was added to the reaction mixture and the yield was increased by about 50%. Further, the yield was increased by 77% against the base EP yield. However, the yield got slightly subdued by the addition of 15 mg ZnAl LDH along with 1 mL 1-hexene. For validation purposes, the polymerization reactions were carried out with t-bu-Zr catalyst. A similar trend of the product yield was observed for the t-bu-Zr catalyst too (Figure 2).

The increase in activity is the attribute of the comonomer presence. The activity enhanced due to different factors, improved solubility of the macro chain in the medium offering high solubility of the monomer gases, higher chain transfer activity, and modification of active center quality [48,49,50]. A similar study was reported by Da Silva et al. [50]., where, the terpolymer of ethylene-propylene-1-pentene showed a higher yield with respect to the homopolymer.

### 3.3. DSC and TGA Result

**Figure 3:**
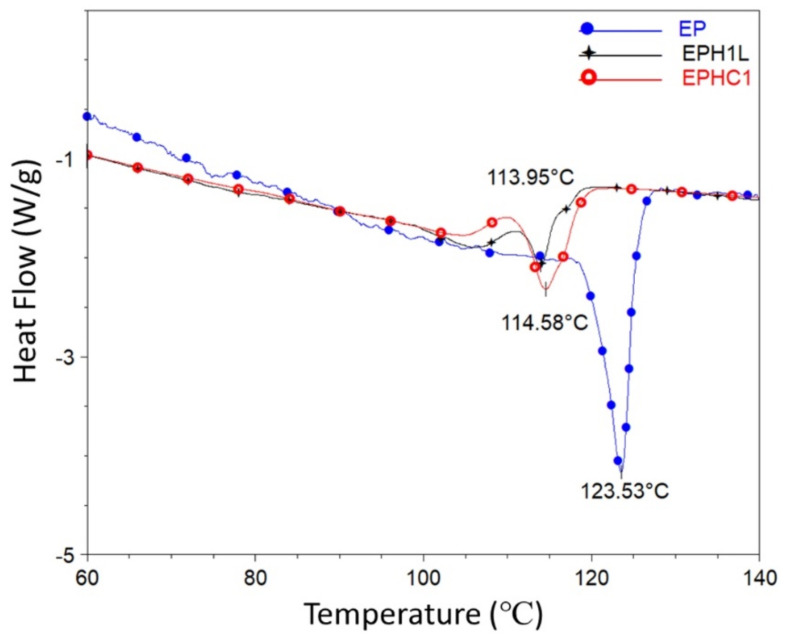
DSC curve of the heating cycle for EP, EPH1, and EPH1L is plotted. The peaks of the corresponding plots are designated as the Tm value of the sample.

The DSC analysis of the polymer samples synthesized by the Zr catalyst was performed. The DSC peaks of different samples are shown in Figure 3. The peak of the DSC heating cycle curve assigns the value of melting temperature (Tm), whereas the peak integral area is associated with the crystallinity of a polymer sample. An intense peak was observed corresponding to the neat EP sample. While terpolymer samples exhibited a comparatively dwarf, broad, and bimodal Tm peak. The in-situ addition of 1-Hexene to the EP polymer shifts the Tm peak towards the left (Figure 3). The Tm values of the samples are listed in Table 2. Increasing 1-Hexene concentration showed a gradual decrease in the crystallinity and the melting temperature of the polymer. These attributes of the terpolymer could be because of the presence of short-chain branching (SCB) or due to enhanced chain transfer activity leading to the formation of low molecular weight and short-chain-branched polymer [48,49].

**Figure 4:**
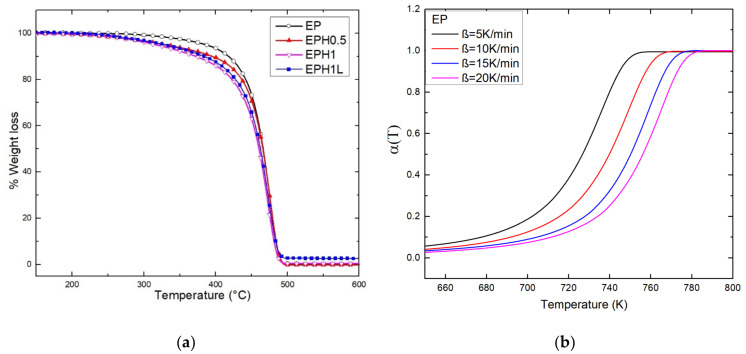
(**a**) TGA plot of the neat EP and terpolymers. (**b**) the plot shows fractional conversion of EP against temperature at different heating rates.

These reasonings also justify the TGA trend for the set of samples. TGA plots (at 10 °C/min heating rate) of neat EP and terpolymer are shown in Figure 4a. The terpolymer undergoes early degradation with respect to the neat EP. It was evident from the TGA plot that the thermal stability of terpolymers was in inverse relation to the amount 1-Hexene added during the in-situ polymerization reaction. The temperature at 10% weight loss (T_0.9_) is mentioned in Table 2. T_0.9_ values for neat-EP, EPH0.5, EPH1 were 418, 395, 372 °C respectively. However, a remarkable result was observed that the EPH1L sample containing only 0.3% ZnAl-DDS LDH showed improved thermal stability by 11.5 °C with a T_0.9_ value equal to 483.5 °C. The incorporated nanomaterial behaves as a thermal barrier and barrier for the diffusion of combustible gases. Therefore, it imparts resistance against thermal degradation and facilitates better thermal stability. Similar results of thermal stability based on the incorporation of nanomaterials such as LDHs and graphene with polymers have been reported in the literature [37,51,52].

The TGA performed at four different heating rates (β = 5, 10, 15, 20 °C/min) were studied. Figure 4b shows the plot of fractional conversion against temperature for EP copolymer. The fractional conversion curve shifts right at higher heating rates. Due to the poor thermal conductivity of the polymer, a higher temperature is required higher heating rates to achieve equivalent fractional conversion. The α versus temperature of terpolymers is available Supplementary Materials as Figure S1.

### 3.4. Kinetic Model Using Theoretical Master Plot

**Figure 5:**
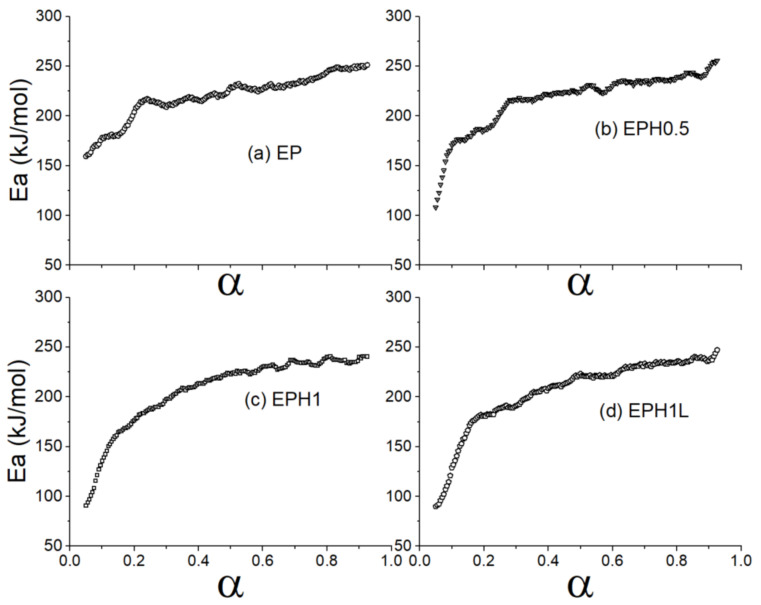
Plots show activation energy of samples against the alpha value (**a**) EP (**b**) EPH0.5 (**c**) EPH1 (**d**) EPH1L.

The Friedman equation (Equation (6)) was applied to evaluate the activation energy against fractional conversion. The terpolymer exhibited a lower activation energy requirement (Figure 5) at the initial stage of the thermal degradation. The Ea values were found to be in inverse relation with the 1-hexene concentration introduced during the in-situ synthesis of the EPH terpolymer. The increasing concentration of 1-hexene added during the in-situ process underwent early thermal degradation. This could be primarily because of the high presence of the branching in the polymer chain caused by the 1-hexene addition. The branches are comparatively easier to break than the straight polymer chains. The lower Ea value could be related to the C-C bond cleavage of the side chains while comparatively higher Ea is required to break long carbon chains [53,54]. However, the activation energy required for the thermal degradation in a range α > 0.2 for the polymer was well high as found in the case of the EP copolymer.

**Figure 6:**
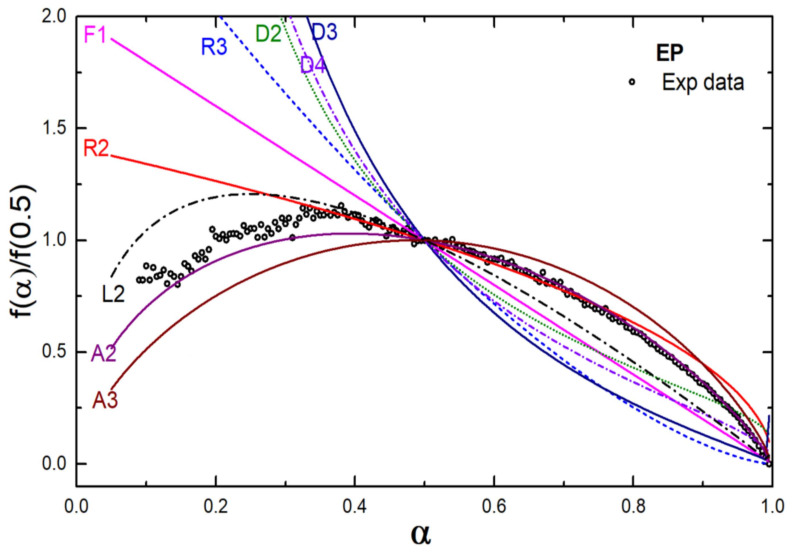
Generalized master plot for the EP copolymer.

The degradation kinetics of the EP and EPH polymers were studied using a generalized master plot technique. A generalized master plot shows a comparison of the theoretical kinetic model (Table 1) with the experimental data, which facilitates insight into the degradation mechanism of a particular polymer. The activation energy evaluated using the Friedman method was utilized to the master plot. The Ea values listed in Table 2 were utilized for the master plot using Equation (10). Figure 6 shows the generalized master plot of the EP copolymer. The plot infers that the EP copolymer is expected to follow a mechanism where the polymer undergoes random nucleation and growth of nuclei through different nucleation sites during the thermal degradation and therefore the degradation is associated with nucleus growth models (Avrami-Erofeev equation, A2 model).

The master plots of terpolymer and its LDH nanocomposite samples (EPH0.5, EPH1, and EPH1L) are shown in Figure S2 (Supplementary Materials). The master plots of the terpolymers indicated that the thermal degradation kinetics of these samples also followed nucleus growth models (Avrami-Erofeev equation, A2 model) but only in a certain range of α (Table 3). The terpolymer and its nanocomposite followed the A2 model in 0.15 < α < 1, the degradation model corresponding to the terpolymers are not clear in the early stages of the degradation i.e., for α < 0.15. The reason for this anomaly could be the very low Ea value during the early stages of the degradation. This could be attributed due to the increase in the number of side branches in the polymer backbone with an increase in the in-situ concentration of the 1-hexene [53,54] added during the polymerization reaction.

**Figure 7:**
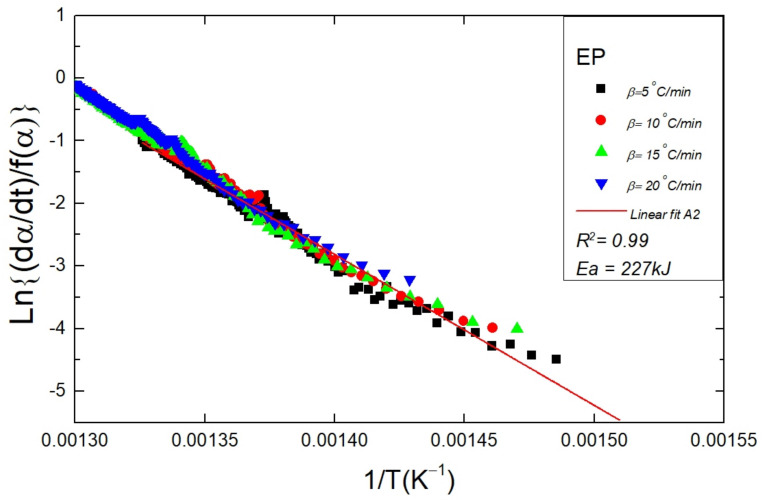
The figure shows the CKA plot for a neat EP sample. The data points obtained at different heating rates (5, 10, 15, 20 °C/min) are fitted to the A2 model using Equation (11). The regression is utilized to estimate the Ea value for a sample.

The confirmation of the degradation kinetics of the polymers is concluded by the goodness of regression in the combined kinetic analysis (CKA) (Equation (11)). The regression coefficient for the A2 mechanism in the CKA plot was found to be high in the given range of α as shown in Figure 7. The activation energy values (Table 3) estimated using the CKA were close to that evaluated through the Friedman method. Therefore, the CKA analysis upholds the master plot outcome, and all the polymer samples followed the A2 mechanism in a certain range of α. The activation energy related to the thermal degradation EP copolymer was estimated using the CKA method which was equal to 227 kJ/mol, this value of Ea is in very close approximation to that reported in our recently published work [36]. To the best of the author’s information, the activation energy for EP copolymer and EPH terpolymer has not been reported in the literature before. However, the activation energy as reported by our research group for a polyethylene (PE) sample was equal to 232 ± 11 kJ/mol [37]. In literature, Ea related to the degradation lies mostly in the range of 200 to 260 kJ/mol [53,54,55]. The variation of the Ea values is due to the dependence of the degradation on the polymer characteristics such as molecular weight, chain length, and branching.

## 4. Conclusions

EP copolymer, EP-1-hexene terpolymer, and its nanocomposite was synthesized by an in-situ process. A remarkable enhancement in the product yield was observed by the addition of 1-hexene during the polymerization reaction. The terpolymer nanocomposite containing only 0.3 weight percent of ZnAl LDH enhanced the thermal stability by two digits. The thermal degradation of the co- and terpolymer was meticulously studied by the Friedman method and Master plot technique. The adopted technique accesses the prediction of the degradation mechanism of the materials.

## Figures and Tables

**Table 1:** Kinetic functions for most commonly used kinetic models. Reprinted (adapted) with permission from Sa’nchez-Jime’nez et al. [34] Copyright 2010 American Chemical Society.

Mechanism	Symbol	f(α)
phase boundary-controlled reaction (contracting area)	R2	${\left(1-\alpha \right)}^{1/2}$
phase boundary-controlled reaction (contracting volume)	R3	${\left(1-\alpha \right)}^{3/2}$
random nucleation followed by an instantaneous growth of nuclei (Avrami-Erofeev equation, n = 1)	F1	$\left(1-\alpha \right)$
random nucleation and growth of nuclei through different nucleation and nucleus growth models (Avrami-Erofeev equation, n ≠1)	An	$n\left(1-\alpha \right)\;{\left[-ln\left(1-\alpha \right)\right]}^{1-1/n}$
two-dimensional diffusion	D2	$\frac{1}{\;\left[-ln\left(1-\alpha \right)\right]}$
three-dimensional diffusion (Jander equation)	D3	$\frac{\;3{\left(1-\alpha \right)}^{3/2}}{2\;\left[1-{\left(1-\alpha \right)}^{\frac{3}{2}}\right]}$
three-dimensional diffusion (Ginstling-Brounshtein equation) 3	D4	$\frac{3}{2\;\left[{\left(1-\alpha \right)}^{-\frac{1}{3}}-1\right]}$
random scission, L = 2	L2	$2\left(\alpha^{1/2\;}-\alpha \right)$
random scission, L >2	Ln	no symbolic solution

**Table 2:** This table shows the melting temperature, crystallinity, temperature at 10% weight loss (T_0.9_), and activation energy of product samples with different chemical compositions.

	Catalyst Name	Zirconocene			
Sample Name	Composition	Tm (°C)	Crystallinity *	T_0.9_ (°C)	Ea ^#^ (kJ/mol)
EP	Neat EP	123.5	58.7	430 ± 2	244
EPH0.5	EP + 0.5 mL 1hexene	113.3	46	395	217
EPH1	EP + 1 mL 1hexene	114.6	26.8	372	207
EPH1L	EP + 1 mL 1hexene + LDH	114	29	383.5	207

* The crystallinity was measured by DSC. **#** The Ea values were measured by the Friedman method.

**Table 3:** Shows the kinetic model followed by samples in range alpha, regression coefficient value, and activation energy value.

Sample	Kinetic Model	α-Range	Ea [kJ/mol]	R^2^
EP	A2	0.05 < α < 1	228	0.99
EPH0.5		0.15 < α < 1	205	0.99
EPH1		0.15 < α < 1	185	0.98
EPH1L		0.18 < α < 1	187	0.986

## Data Availability

Data available on request.

## Supplementary Materials

The following supporting information can be downloaded at: https://www.mdpi.com/article/10.3390/polym14030634/s1, Figure S1: Fractional conversion (α) with respect to temperature is plotted for (a) EPH0.5 (b)EPH1 (c) EPH1L; Figure S2: Shows the Generalized master plot and Combined kinetic (CKA) plot (a) EPH0.5 (b)EPH1 (c) EPH1L.
