# Supplementary material for: Thermal Degradation Kinetics Analysis of Ethylene-Propylene Copolymer and EP-1-Hexene Terpolymer
Source: Polymers (Basel). 2022 Feb 7;14(3):634. doi: 10.3390/polym14030634 (PMC8839618; doi:10.3390/polym14030634)
Supplement: Supplementary file 1 [file polymers-14-00634-s001.zip › polymers-1519460-supplementary.pdf]

polymers

MDPI

Supporting Information

Thermal Degradation Kinetics Analysis of Ethylene-Propylene Copolymer and EP-1-Hexene Terpolymer

Hassam Mazhar [1], Farrukh Shehzad [1], Sung-Gil Hong [2] and Mamdouh A. Al-Harthi [1,3,*]

1. Department of Chemical Engineering, King Fahd University of Petroleum & Minerals, Dhahran 31261, Saudi Arabia; hassam.mazharulhaque@kfupm.edu.sa (H.M.); farrukh@kfupm.edu.sa (F.S.)
2. TS&D Center, S-OIL Corporation, Seoul 07793, Korea; sghong0927soil@gmail.com
3. Center for Refining and Advance Chemicals, The Research Institute, King Fahd University of Petroleum & Minerals, Dhahran 31261, Saudi Arabia

* Correspondence: mamdouh@kfupm.edu.sa

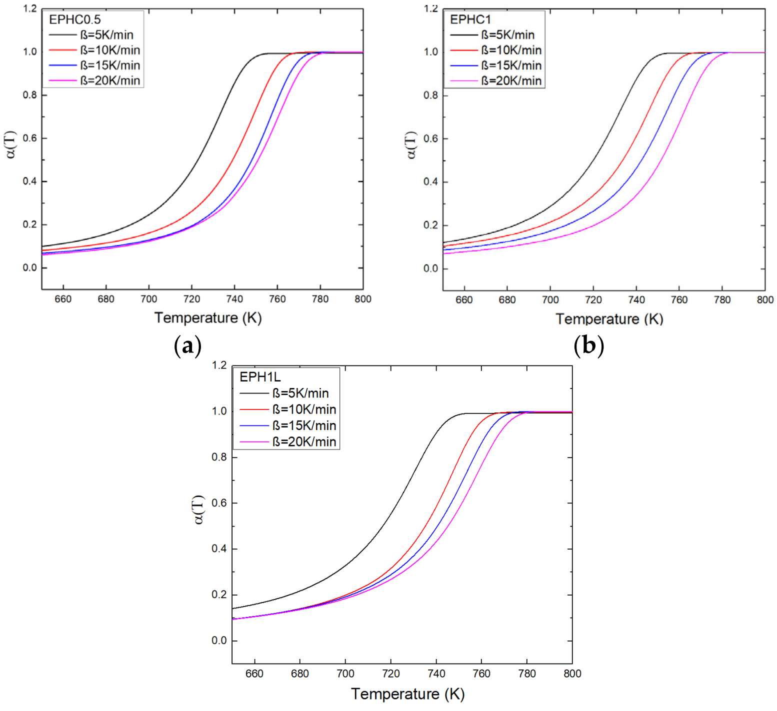

(c)

Figure S1. Fractional conversion (α) with respect to temperature is plotted for (**a**) EPH0.5 (**b**)EPH1 (**c**) EPH1L

Master plot CKA plot

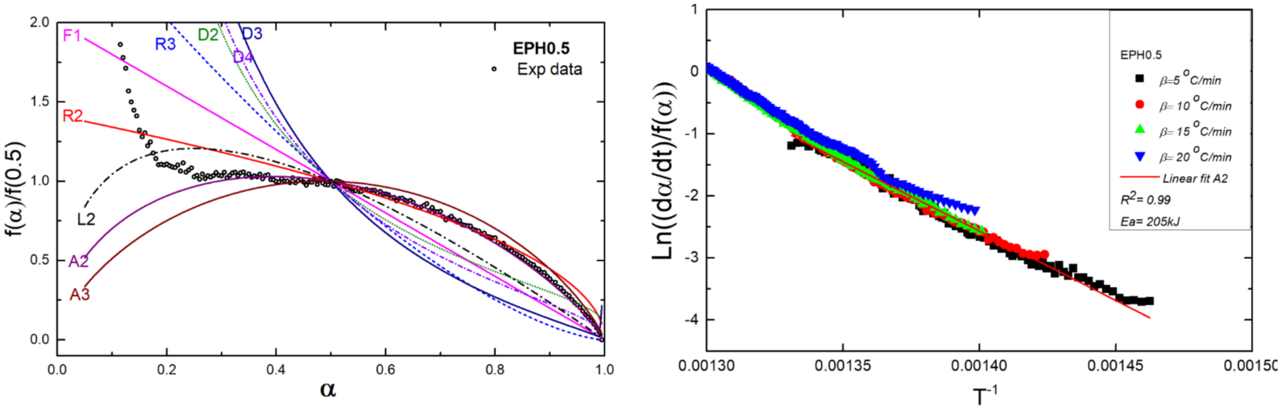

(a)

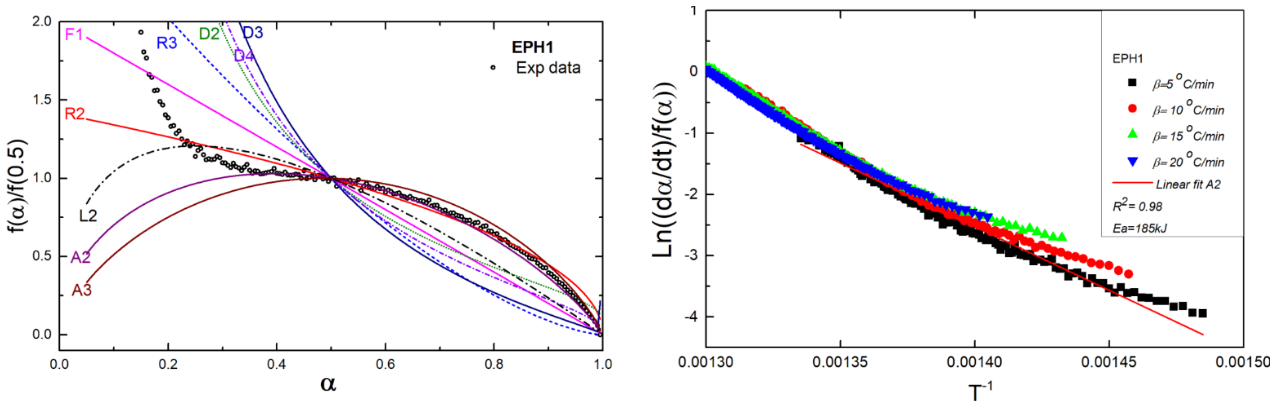

(b)

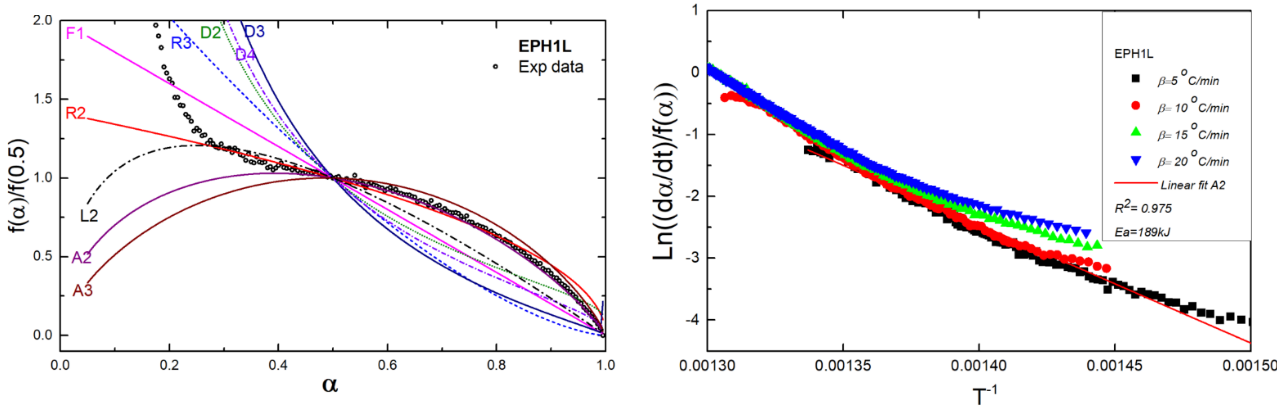

(c)

Figure S2. : Shows the Generalized master plot and Combined kinetic (CKA) plot (**a**) EPH0.5 (**b**)EPH1 (**c**) EPH1L.
